# Principal Postulates of Centrosomal Biology. Version 2020

**DOI:** 10.3390/cells9102156

**Published:** 2020-09-24

**Authors:** Rustem E. Uzbekov, Tomer Avidor-Reiss

**Affiliations:** 1 Faculté de Médecine, Université de Tours, 10, Boulevard Tonnellé, 37032 Tours, France; 2 Faculty of Bioengineering and Bioinformatics, Moscow State University, Leninskye Gory 73, 119992 Moscow, Russia; 3 Department of Biological Sciences, University of Toledo, 3050 W. Towerview Blvd., Toledo, OH 43606, USA; Tomer.AvidorReiss@utoledo.edu; 4 Department of Urology, College of Medicine and Life Sciences, University of Toledo, Toledo, OH 43607, USA

**Keywords:** centrosome, centriole, cilia, flagella, microtubules

## Abstract

The centrosome, which consists of two centrioles surrounded by pericentriolar material, is a unique structure that has retained its main features in organisms of various taxonomic groups from unicellular algae to mammals over one billion years of evolution. In addition to the most noticeable function of organizing the microtubule system in mitosis and interphase, the centrosome performs many other cell functions. In particular, centrioles are the basis for the formation of sensitive primary cilia and motile cilia and flagella. Another principal function of centrosomes is the concentration in one place of regulatory proteins responsible for the cell’s progression along the cell cycle. Despite the existing exceptions, the functioning of the centrosome is subject to general principles, which are discussed in this review.

## 1. Introduction

Nearly 150 years ago, almost simultaneously, three researchers described in dividing cells two symmetrically located structures that looked like a “radiance” and were called the centrosphere [1,2,3]. At the centrosphere’s focus, granules were sometimes visible, which were originally called “polar corpuscles” [3]. Van Beneden and Nate [4] and independently Boveri [5] found that the polar corpuscles do not entirely disappear after mitosis, but remain in interphase, often located near the geometric center of the cell.

Later, these granules were named centrioles [6]. Shortly after, Henneguy and von Lenhossék showed that centrioles and basal bodies are the same structure at distinct functional stages. They proposed a “hypothesis about the homology of centrioles and basal bodies of flagella” [7,8]. This idea was overlooked for many years but ultimately is correct. After the ultrastructure of the centrosome was studied using transmission electron microscopy, centrioles (and basal bodies) were shown to have a conserved structure in many types of cells—hollow cylinders containing triplets of microtubules (MTs) in their walls [9,10,11]. The unique centrally symmetric structure of centrioles has always generated amazing, and sometimes fantastic, hypotheses about the centrosome’s origin and functions. Fundamental advances in molecular biology, immunocytochemistry, and high-resolution light microscopy have allowed us to advance the understanding of the fundamental functions of this organelle that is small in size, but most important for many functions in the cells.

Summing up the many years of centrosome research, we formulated 12 postulates of centrosomal biology in 2007 [12]. A few years later, these postulates were somewhat supplemented and developed in a book on the centrosome [13]. The discoveries of recent years make it necessary once again to return to these postulates and update them.

## 2. Postulates of Centrosomal Biology

### 2.1. The Centriole and the Basal Body Are Two Forms of the Same Organelle

**Figure 1:**
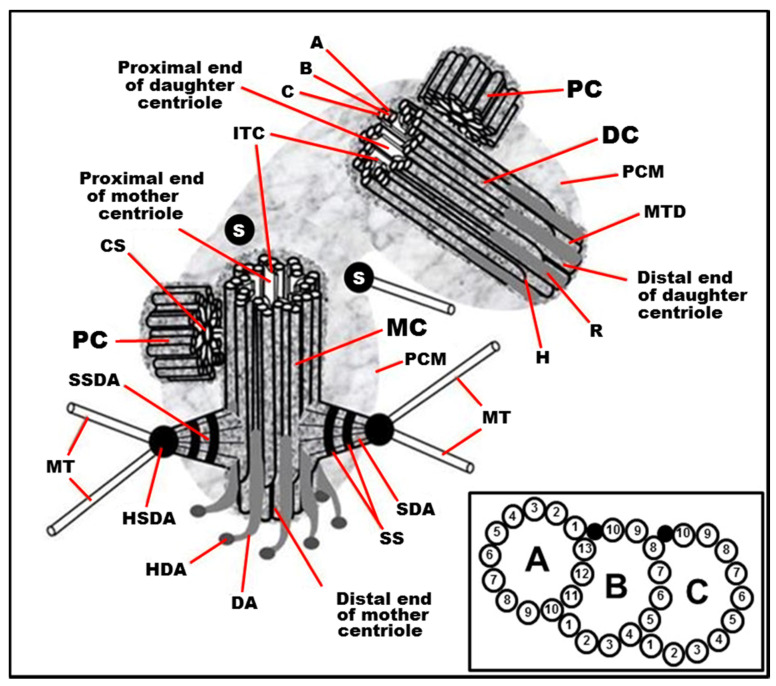
Typical centrosome structure in the S phase of the cell cycle in proliferating mammalian cells. MC: mother (mature) centriole; DC: daughter centriole; PC: procentriole; PCM: pericentriolar material (pericentriolar matrix); A: “A” MT of triplet; B: “B” MT of triplet; C: “C” MT of triplet; H: hook of “C” MT; MTD: A-B MT duplex (in the distal part of centriolar cylinder); ITC: internal triplets connections system (scaffold structure), which include A-C linkers; CS: cartwheel structure (an axis with spokes); SDA: sub-distal appendage; HSDA: head of sub-distal appendage; SSDA: the stem of sub-distal appendage (connected to three triplets in this case); S: satellites; SS: the striated structure of sub-distal appendage stem; MT: microtubule; DA: distal appendage; HAD: head of distal appendage; R: rib. From [12] with modifications. Insertion: The fine ultrastructure of the MT triplet, showing protofilaments of MTs (the data from [22,23] were used to make this drawing).

The centrosome usually organizes and orients the mitotic spindle. The basal body nucleates and anchors the cilium in interphase. The centrosome consists of two centrioles surrounded by pericentriolar material after mitosis in the G1 phase or the G0 phase of the cell cycle. At the end of the interphase before the next mitosis in the G2 phase of the cell cycle, the cell has two centrosomes, each having two centriolar cylinders in its composition. Each centriole consists of 9 MT triplets, and it is associated with subdistal appendages, distal appendages, striated roots, and satellites (Figure 1). In some cases or certain stages of the development of organisms, the walls of the centrioles may contain MT doublets [14,15,16], MT singlets [17,18,19], or no MT at all [20,21], while maintaining nine-beam symmetry.

The structure of centrioles during the formation of cilia or flagella is conserved in the majority organisms and cell types—9 MT triplets (aka “9×3”) with no central MTs (aka “+0”) that is summarized by the formula “9×3 + 0”. In rare cases, cilia appear to form a basal body consisting of doublets MTs [24,25,26]. The MT triplets are made three tubules named the MT “A”, the MT “B”, and the MT C”. Only MT “A” has a closed rounded shape on a transverse section and consists of 13 protofilaments, as most cellular MTs [27,28]. MT “B” has the shape of an arc adjacent to MT “A” and having three or four common protofilaments with it. MT “C” also has the shape of an arc adjacent to MT “B” and has three or four common protofilaments with it. The number of protofilaments in MT “B” and MT “C” can be 10 or 11 in centrioles from different organisms, and the total number of protofilaments in one triplet can thus be from 33 to 35 [10,28,29,30,31,32]. There are suggestions that protofilaments number 11 of MT “B” and MT “C” may have a special biochemical composition [28].

The centriolar cylinders’ central nine-fold symmetry is established during the formation of the “cartwheel” structure [10,33]. This symmetry is generated by lateral interaction of the N-terminal domains of SAS-6 protein dimers [34,35]. The cartwheel structure disassembles in mature centrioles as cells exit mitosis of vertebrates [33,36] but remains in mature centrioles of insects [37].

### 2.2. The Centriole Is a Polar Structure with Two Morphologically and Functionally Different Ends

The centriole’s distal end (where the plus ends of the MT are) can serve as the site of cilia formation, and it associates with distal appendages. A complex of proteins, including CP110 and CEP97, is found at this end and supports cilium formation [38].

Subdistal appendages are also more often formed closer to the distal end of the centriole. The number of distal appendages (for centrioles of centrosome) and their homologous structures in basal bodies—alar sheets—are always equal to nine [39,40]. The number of subdistal appendages (the first original name of these structures was pericentriolar satellites [41]) is variable for different types of cells and various conditions and ranges from 0 to 13 [42,43,44].

The foot is the homologous structure of subdistal appendage in the basal body. One to two feet are found per basal body, and they are located closer to their distal end [40,45,46]. The foot base is connected to two or three triplets in a basal body [47]. In the basal bodies of functionally active cilia, feet are located in the plane of the cilia beat [48].

New centriolar cylinders (procentrioles) usually appear perpendicular to the mother centriole’s surface closer to its proximal end [33], where the minus ends of the MT triplets are located.

The pericentriolar material can also be located asymmetrically, surrounding only the centriole proximal part [36,49]. The MT “A” centriole’s proximal end is covered with a conical structure with a morphology similar to the γ-tubulin ring complex (γ-TuRC) [50].

The polarity of the centriole is manifested in the orientation of some of its components. When viewed from the proximal end of the centriole, the MT triplets are always twisted counterclockwise (in the direction from the inner MT “A” to the outer MT “C”), and the distal appendages are always twisted clockwise [12,51] (Figure 1).

### 2.3. The Outer Diameter of the Distal Part of the Centriolar Cylinder Is Smaller Than That of the Proximal End

There are two reasons for the diameter differences between the centriole ends. (i) The MT triplets transform into doublets of MT as MT “C” is usually shorter than MT “A” and MT “B”. (ii) The angle of inclination of the MT triplets (the line passing through the centers MT “A” and MT “B”) to the radius of the centrioles at the distal end of the centrioles is 80–90 degrees (the doublets lie almost in a circle). At the proximal end of the centriole, the triplets’ inclination to the radius is 50–55 degrees (the triplets are deployed like turbine blades). The inner radius of the lumen (the distance from the center of the centriole to MT “A”) does not change. In contrast, the outer radius (the distance from the center of the centriole to MT “C”) increases due to the unfolding of the triplets [36]. This twist’s angle appears to be controlled by the A-C linkers in the proximal segment and an inner scaffold at the distal portion [52].

### 2.4. The Centriolar Cylinder Length Is Highly Regulated

The centriolar cylinder’s length is precisely set and usually ranges from 200 to 700 nm depending on cell type and cell cycle phase [53,54]. However, in some cell types, extremely long centrioles can be observed [10,55,56]. The growth of centrioles in length is regulated by a complex of proteins, including SAS4/CPAP, POC1, and POC5 [57,58,59,60]. Overexpression of CPAP or its interaction partners, CEP120 and SPICE1 (Spindle and Centriole Associated Protein 1), lead to the assembly of excessively long centrioles. The protein CP110 is an antagonist to the CPAP; CP110 caps the distal end of the growing centriole [61,62,63,64].

### 2.5. In the Centrosome of Proliferating Cells, Two Centrioles Differ Structurally and Functionally

Only the older (mother) centriole has appendages at the distal end. Only the mother centriole has subdistal appendages. MT nucleating centers are located predominantly on or near the mother centriole. Gamma-tubulin [65,66] is the basis of two types of protein complexes that nucleate MTs—the large Gamma-TuRC [67] and small Gamma-TuSC [68,69]. There are other protein complexes on the centrosome that anchor the MTs. These complexes include CAP 350/FOP/EB1 [70], PCM/BBS4/ninein/centrin/pericentrin [71], and ninein/ODF2/Cep170/centriolin/epsilon-tubulin [72]. MT nucleation and MT attachment activities are located in the subdistal appendages’ heads, on the surface of the centrioles, and in the pericentriolar material. Centriolar satellites are dense rounded structures containing protein complexes of the pericentriolar material located near centrioles and undergo cell cycle-dependent assembly and disassembly [73,74,75,76]. They move towards the centrosome along MTs in a dynein-dependent manner; participate in targeting of centrin, pericentrin, and ninein to the centrosome; and are implicated in ciliogenesis [75,77,78].

### 2.6. The Proximal Ends of the Mother and Daughter Centrioles Are Connected via a Bundle of Thin Fibers

The Proximal Ends of the Mother and Daughter Centrioles are Connected Via A Bundle of Thin Fibers [79,80,81]. The composition of this ligament includes proteins rootletin, beta-catenin, and C-NAP1 [82,83,84]. Severing this connection can lead to centriole separation. The regulation of this separation is controlled by Nek2 kinase [84]. Separation of mother and daughter centrioles after mitosis is a necessary prerequisite for the start of centrioles duplication [85].

The separation of the mother and daughter centrioles in G1 phase of the cell cycle is a different process from the separation and divergence of the two centrosomes before mitosis. The last process differently depends on the intactness of MTs and actin microfilaments [86] and is controlled by AuroraA [87,88,89,90], p34cdc2 [91], and Plk1 [92,93,94] kinases. In the process of centrosome separation and movement during the formation of the two poles of the spindle, several types of motors are involved: the cytoplasmic dynein–dynactin–NUMA complex [95,96,97], Xklp2 [98], kinesin-related motors pEg5 [89,91,99], and XCTK2 [100].

### 2.7. The Centriole Lumen Helps in Stabilizing the Centriole

Centriolar MTs have much greater stability than spindle or interphase cytosolic MTs. They are not depolymerized either by anti-MT drugs or by exposure to cold [101,102,103]. However, MT triplets can be disassembled by high (1–2 M NaCl or KCl) salt concentrations once centrioles are isolated [104]. This stability of MT centrioles is associated with stabilizing proteins such as tektins between microtubules [105,106], post-translational polyglutamylation of centriolar tubulin [107,108], and presence of scaffold structures inside the centriole lumen.

Inside the centriolar cylinder, an interconnected system of ligaments connects the MT triplets [10,29,32,39,73]. The structure of these connections changes from the proximal end to the distal one. MT “A” has a connective with MT “C” of the neighboring triplet. In addition, inward directions from each MT “A” are strands of electron-dense material that are interconnected, with MT “A” and A–C bundles. At the ultrastructural level, the centriolar cylinder’s lumen looks “empty” near the proximal end and filled with electron-dense material at the distal lumen. The protein composition of the A–C linker is unknown. The distal lumen scaffold is made of POC1B, POC5, FAM161A, CETN, and WDR90 [52,109]. Mutating some of these protein results in destabilization of the centriole cylinder [110].

### 2.8. Maintaining a Cylindrical Shape of Centrioles Can Be Independent of MT Triplets

**Figure 2:**
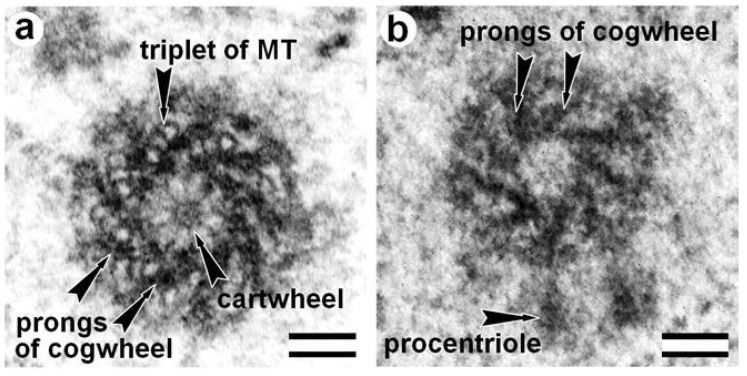
Centriole structure in larvae of two wasps: (**a**) larvae of Nasonia vitripennis, where prongs of cogwheel structure are visible between triplets of MT; and (**b**) early larvae of *Anisopteromalus calandrae*, where the centriole has a cogwheel without MT. The cartwheel structure is not clearly visible in the centriole lumen. View from the distal end of the centriole. Scale bar: 100 nm. From [20] with modifications.

Centrioles do not have MTs in haploid male larvae trophocytes and hypodermal cells of wasps *Anisopteromalus calandrae* [20]. However, their MT-free centrioles’ shape and size are similar to canonical centrioles with MT triplets of adult (imago) insects or late larvae of different wasps’ types (Figure 2). The centriole’s symmetry is maintained, and they contain nine prongs of electron-dense material in their walls forming the structure of the “cogwheel” [20]. One possibility is that at this stage of development, the genes encoding the proteins responsible for the construction of MT triplets have not yet turned on. Alternatively, the MTs disassembled after the centriole formed. Practically identical cogwheel structure without MTs triplets was also found in the base of mature spermatozoa flagella of wasps *Cotesia congregata*. It replaced the “normal” centriole during spermiogenesis, which is present in spermatids [21]. Prongs of cogwheel structure are visible between triplets or doublets of MT in centrioles of other insects, particularly in *Drosophila*. It was shown that the protein SAS4 [111] is concentrated in these regions.

**Figure 3:**
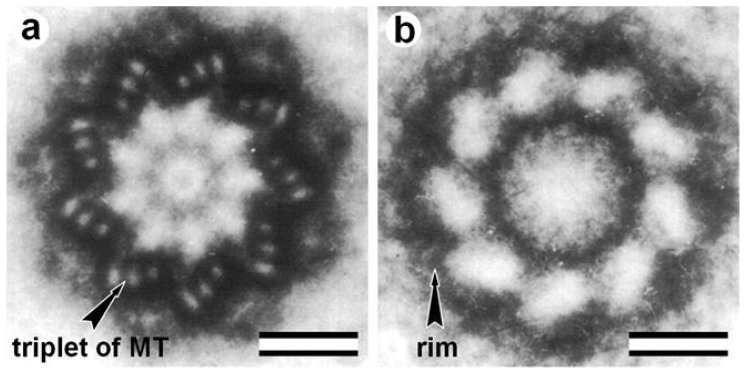
Rotation images of centriole and centriolar rim. Nine photographs with a rotation angle of 40° were superimposed to obtain images: (**a**) centriole; and (**b**) centriolar rim after 1 M KC1 treatment. Scale bar: 100 nm. From [104] with modifications.

After processing the isolated centrioles from bovines spleen by 2 M salts, the triplets’ MTs are completely disassembled [104]. Holes were found at the MT sites (Figure 3); still, the centrioles’ cylindrical shape is preserved. The authors called the electron-dense structure surrounding triplets “centriolar rim” [104]. Later, was reported that the PCM is not homogeneous and that some centrosomal proteins are closely associated with the centriole to form a distinct PCM compartment called the “PCM tube”. In contrast, other proteins are more peripheral [112,113]. These MT surrounding protein complexes help maintain the cylindrical shape and provide mechanical strength in MT’s absence.

### 2.9. The Structure and Activity of Centrosomes Differ in Interphase and Mitotic Cells

The subdistal appendages and primary cilia disappear [36,114], and an amorphous mitotic halo surrounds the centrosome in mitosis. In addition, the centrosome-associated interphase MTs are completely depolymerized before mitosis. Finally, mitotic MT asters form around each of the two centrosomes during prophase. These MTs, together with other MTs nucleating activities, organize and form the mitotic spindle in metaphase [78]. All mitotic MTs have their minus ends near the centrosome but have three options for localizing their plus end: (1) MT plus end is directed in the opposite direction from the chromosomes and is localized near the cell membrane—astral MTs; (2) MT plus end is associated with the kinetochore of chromosomes—kinetochore MTs; and (3) MT plus end interacts with an MT coming from the opposite pole of the spindle—interzonal MTs. The halo also contains many short MTs, which probably later transform into one of three types. Mitotic MTs have dynamic and biochemical characteristics different from interphase MTs; in particular, they are less resistant to anti-microtubule drugs [115]. The ability of centrosomes to nucleate MT in mitosis increases several times during preparation to cell division (G2 phase of the cell cycle) in a process named centrosome maturation [36,116,117].

### 2.10. New Centrioles Are Usually Formed in Association with Mother Centrioles but Can Be Formed without Preexisting Centriole De Novo

**Figure 4:**
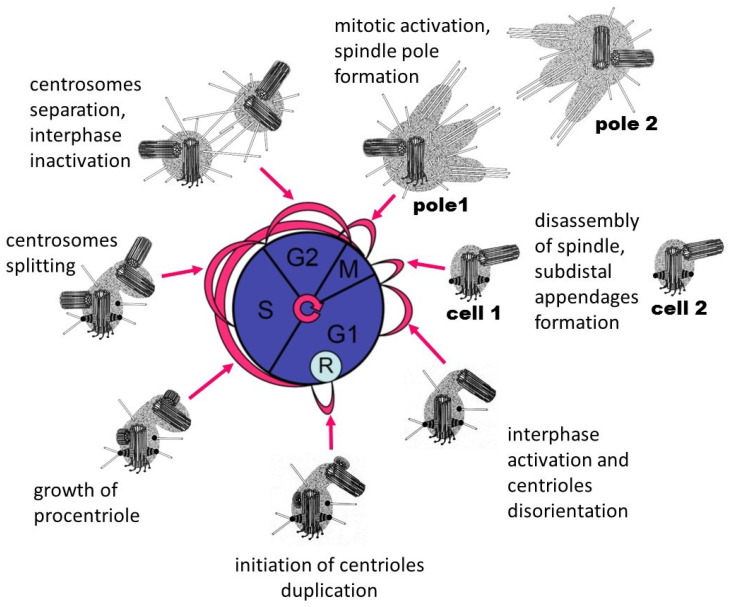
The relationship of the cell (nuclear) cycle and centriolar cycle. R, restriction point.

Duplication of centrioles occurs only one time per cell cycle; on each mature centriole, only one procentriole appears (Figure 4). The accuracy of regulation of these processes is controlled by a complex of proteins that were first identified in *Caenorhabditis elegans*: ZYG-1 [118,119], SPD-2 [19,120,121], SAS-4 [122,123], SAS-5 [124], and SAS-6 [125,126]. In human cells, the ZYG-1 homolog is PLK4 kinase [127]; in Drosophila cells, it is SAK/PLK4 kinase [128]. More than 30 proteins have already been described that are somehow involved in the duplication of centrioles [129]. In mammals and insects, centriole duplication starts by CEP152/Asterless that recruits PLK4 [130,131,132]. CEP152 form a ring together with CEP63 and CEP57 around the proximal part of the mother centriole [133,134].

Centriole duplication is independent of DNA replication [135,136,137] and starts near the end of the G1 phase (Figure 4) of the cell cycle [138,139]. At this time, a complex of biochemical reactions in the cell is launched dependent on the cell’s size, the presence of external growth factors, the growth conditions of the cell, and its interaction with surrounding cells [140,141,142]. It has been shown that the Cyclin D/CDK 4/6 complex phosphorylates the pRB protein, which loses its ability to bind the transcription activation factor EF2. The released transcription factor EF2 activates the synthesis of Cyclin E and Cyclin A, which starts the process of duplication of centrioles. Thus, DNA replication and centriole duplication are regulated by a single cytoplasmic mechanism. However, centriole duplication begins earlier than DNA replication [138], suggesting additional regulatory mechanisms.

In some cases, new centrioles form without preexisting centrioles. In the ciliary epithelium cells, which have hundreds of cilia, the centrioles form via deuterostomes [46,143]. Deuterosomes can be assembled autonomously from parental centrioles by *de novo* centriole amplification in multiciliated cells [144,145]. In murine early embryonic development, centrioles appear *de novo*, but the mechanism is unclear [146]. In cells with centrioles, centrioles elimination by micro-irradiation does not prevent procentriole formation at S phase. In contrast, in this case, many new centrioles are formed during the S phase [147]. In addition, centrioles can appear without progenitors during parthenogenetic development [148].

These observations suggest that centrioles can assemble independently of preexisting centrioles, and the role of centriole duplication is to restrict the number of assembling centrioles to only one new centriole.

### 2.11. The Centrosomes Have Four Types of MT Nucleating Activity

Many of the centrosome’s critical function is mediated by MTs that are nucleated by it at different cell types and cell cycle phases. This includes four main MTs nucleating activities:

(1) Formation of two types of interphase MTs: (i) a radial MTs system around the centriole; and (ii) non-centrosomal (free) cytoplasmic MTs that were polymerized on the centrosome and later released to the cytoplasm.
(2) Formation of three types of mitotic spindle microtubules: (i) astral MTs; (ii) kinetochore MTs; and (iii) interzonal MTs.
(3) Formation of MT of procentrioles.
(4) Formation MTs of cilia or flagellum, or a related structure, known as the centriolar adjunct, that is found in mammalian spermatids [149,150].

**Figure 5:**
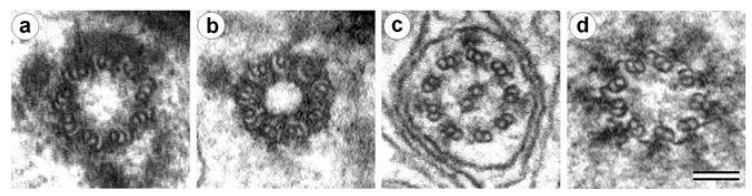
Four types of MT-contained structures: (**a**) proximal centriole in the early pig spermatid—formula “9×3 + 0”; (**b**) centriolar adjunct in the early pig spermatid—formula “9×2.5 + 0”; (**c**) flagellum in early pig spermatid—formula “9×2 + 2”; and (**d**) the cilia-like structure structurally similar to the primary cilium of mammals in the spermatid of the wasp *Anisopteromalus calandrae*—formula “9×2 + 0”. Scale bar: (**a**–**d**) 100 nm. (**a**–**c**) from [150]; and (**d**) from [20].

The more mature mother centrioles usually form the primary cilia with the formula 9×2 + 0 (Figure 5). The centrioles that arose in the current cell cycle and therefore are more immature form the motile cilia. Motile cilia have the formula 9×2 + 2 with the nine doublets MTs of the wall and two central MTs (Figure 4). The mature (proximal) centriole forms a centriolar adjunct in mammalian spermatids [150,151]. The centriolar adjunct has a formula similar to the primary cilium (9×2.5 + 0) (Figure 5). Simultaneously, the centriolar adjunct forms in the spermatid, the daughter (distal) centriole form a motile flagellum with the formula 9×2 + 2 (Figure 5).

### 2.12. The Complete Process of Centrioles Maturation from Procentriole to Mother Centriole Takes More Than One and a Half Cell Cycles in Duration

The complete process of centrioles maturation from procentriole to mother centriole takes more than one and a half cell cycles in duration [36]. The exact timing of procentriole initiation is debatable [152]. The percentage of cells with procentrioles significantly exceeded the S-phase percentage in the cell cycle in synchronized HeLa cells. This difference may indicate that the process of procentriole formation began before DNA replication start [153]. It was shown later that the initiation of centriole duplication could occur even in the absence of a nucleus in cytoplasts and enucleated sea urchin zygotes [137,154]. Procentrioles were found near mother centrioles two hours before the start of DNA replication in pig kidney cell line, suggesting the procentrioles start to form before DNA replication [138,139]. However, whether this observation is universal in other cell types is unknown.

After its initial appearance, the centriole grows gradually to the mother’s size during the S phase and G2 phase of the cell cycle (Figure 4). It becomes a daughter centriole after mitosis in the newly formed cell. The centriole becomes a mature mother centriole, acquiring a complete set of its cell activities after the second mitosis in its life [36,152]. The cell (nuclear) cycle and the centriolar (centrosomal) cycle are mutually coordinated (Figure 4) at least at two critical points in the cell cycle: the end of the G1 and the end of the G2 phase [13,155]. In addition, some critical events take place in mitosis during a process termed “centriole to centrosome conversion” [156]. Moreover, many proteins involved in the regulation of the cell cycle are concentrated in the centrosome. Interference with this regulation leads to perturbation of cell cycle progression, ultimately leading to overproliferation or degeneration [157].

### 2.13. The Centrosome Is the Center of the Organization of Actin Microfilaments in the Cell

This novel function, “actin organization center”, is in addition to the classic centriole function in MT organizing [158]. The centrosome occupies a central position in the cytoplasm of many types of cells. This position is associated with other interconnected cytoskeleton elements, such as actin microfilaments. This position is regulated by the balance of the tension forces associated with the cytoplasmic dynein [159] and the repulsive forces caused by the growth of MT [160,161]. In addition, the centrosome position depends on the interaction of centrosomal MTs with the actomyosin complex, and on the activity of the actomyosin complex itself [162,163,164]. Besides this, the intermediate filaments’ architecture is indirectly dependent on the centrosome since the intermediate filaments system collapsed during the depolymerization of MT by colchicine or nocodazole [165,166].

### 2.14. Centrioles Can Gain an Atypical Structure and Composition and Become Undetected Using Standard Expectations and Techniques

Centrioles are remodeled and earn distinct novel structures in a species-specific manner that makes them difficult to detect [167,168]. In some insects, the sperm atypical centrioles are lacking MT [25,169,170]. In contrast, in human and bovine sperm, the MTs are present, but they are splayed around two novel rod structures [170]. Like canonical centrioles, these atypical centrioles recruit PCM, form centrosomes and asters, and participate in spindle formation in the zygote. In addition, canonical centrioles become undetected, including during myogenesis, oogenesis, and mice spermatogenesis [171,172,173]. In amoeboflagellate *Naegleria*, centriolar cylinders are formed *de novo* during the transition from centriole-less amoebae to flagellates with basal bodies from precursor complexes that act as radiometry centrioles [174,175]. Therefore, centrioles can gain novel structure and composition while performing many of their classical functions.

### 2.15. The Centrosome Is a Polyfunctional, Multi-Protein, and Cell Regulation Complex; Some of Its Proteins Simultaneously Regulate Several Intracellular Processes

**Figure 6:**
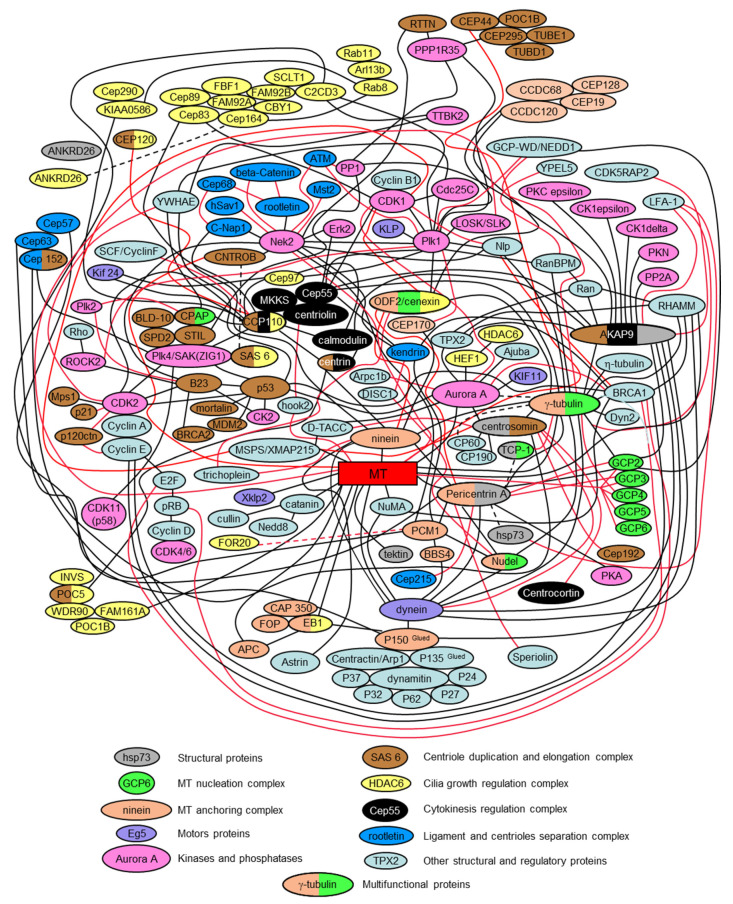
Scheme of protein interactions and functional protein complexes in centrosome (interactome) ([13] with modifications). Red and black lines mark centrosomal protein interactions.

The presented interactome scheme in Figure 6 is only a partial depiction of all centrosomal proteins and their interactions. It is intended to be a simplified diagram that helps to assess the complexity of the centrosome’s biochemical organization. Indeed, each year, more and more centrosomal proteins are characterized. These proteins include proteins that mediate or regulate processes such as signaling pathways, nucleation and anchoring of microtubules, centriole duplication, separation of the daughter centriole from the mother centriole, conversation of centrioles to basal bodies, and nucleating of cilia and flagella. It is often impossible to separate the functions performed by the same protein in different aspects of centrosome functioning.

A single universal classification for centrosomal proteins is unsubtle since they can be organized according to several parameters. Many of them have multiple characteristics for any parameters such as localization, timing, and activity. In the case of localization, some proteins, such as tubulins, are both in the centrioles and pericentriolar material, or the cilium axoneme. In terms of timing, some proteins always present in the centrosome. In contrast, other proteins appear in the centrosome only at specific periods of the cell cycle or at a particular cell differentiation stage. In terms of activity, the centrosomal proteins can be classified according to their biochemical activity—kinases, phosphatases, motors, and structural proteins.

Centrosomal proteins do not exist on their own, but form complex, often interconnected, functional complexes. Figure 6 shows how some of these complexes are related to each other.

The following publications were used to prepare the presented interactome version: [13,19,38,49,52,57,63,68,69,70,71,75,83,84,89,92,93,98,126,127,130,133,134,141,176,177,178,179,180,181,182,183,184,185,186,187,188,189,190,191,192,193,194,195,196,197,198,199,200,201,202,203,204,205,206,207,208,209,210,211,212,213,214,215,216,217,218,219,220,221,222,223,224,225,226,227,228,229,230,231,232,233,234,235,236,237,238,239,240,241,242,243,244,245,246,247,248,249,250,251,252,253,254,255,256,257,258,259,260,261,262,263,264,265,266,267,268,269,270,271,272,273,274,275,276,277,278,279,280,281,282,283,284,285,286,287,288,289,290,291,292,293,294,295,296,297,298,299,300,301,302,303,304,305,306,307,308,309,310,311,312,313,314,315,316,317,318,319,320,321,322,323,324,325,326,327,328,329,330,331,332,333,334,335,336,337,338,339,340,341,342,343,344,345,346,347,348,349,350,351,352,353,354,355].

## 3. Questions and Perspectives

The centrosome continues to remain “the central enigma of cell biology” [30], although many data have been obtained in recent years on many of its various aspects. The exact time of the onset of centriole duplication in the cell cycle and the temporal relationship of this process with DNA replication remains unclear. It is also not entirely clear to what extent the principles of centriole biology are maintained in various cell types. For example, there is no certainty about the mechanism of formation of centrioles in the early development of mammals: How is the only proximal centriole of the spermatozoon transformed into four centrioles at two poles of mitotic division at later stages of development? What is the nature of the differences between this process in mice and other mammals [356]? What are the evolutionary and molecular mechanisms underlying these centriole specific properties [357]? Many of these aspects will be clarified in the near future, and the role of the centrosome, as the main regulatory center of the cell—a kind of “cell processor” [358]—will become more evident.
